# From the Eye of the Albatrosses: A Bird-Borne Camera Shows an Association between Albatrosses and a Killer Whale in the Southern Ocean

**DOI:** 10.1371/journal.pone.0007322

**Published:** 2009-10-07

**Authors:** Kentaro Q. Sakamoto, Akinori Takahashi, Takashi Iwata, Philip N. Trathan

**Affiliations:** 1 Graduate School of Veterinary Medicine, Hokkaido University, Sapporo, Japan; 2 Department of Polar Science, The Graduate University for Advanced Studies, National Institute of Polar Research, Tachikawa, Tokyo, Japan; 3 British Antarctic Survey, Natural Environment Research Council, High Cross, Cambridge, United Kingdom; University of Alabama, United States of America

## Abstract

Albatrosses fly many hundreds of kilometers across the open ocean to find and feed upon their prey. Despite the growing number of studies concerning their foraging behaviour, relatively little is known about how albatrosses actually locate their prey. Here, we present our results from the first deployments of a combined animal-borne camera and depth data logger on free-ranging black-browed albatrosses (*Thalassarche melanophrys*). The still images recorded from these cameras showed that some albatrosses actively followed a killer whale (*Orcinus orca*), possibly to feed on food scraps left by this diving predator. The camera images together with the depth profiles showed that the birds dived only occasionally, but that they actively dived when other birds or the killer whale were present. This association with diving predators or other birds may partially explain how albatrosses find their prey more efficiently in the apparently ‘featureless’ ocean, with a minimal requirement for energetically costly diving or landing activities.

## Introduction

Albatrosses are one of the most vulnerable groups of seabirds consequent on recent natural and anthropogenically-induced changes in the marine environment [1]. They may fly many hundreds of kilometers in just a few days in order to locate their food in the open ocean. Although their diets, foraging movements and fisheries interactions have been studied extensively in recent years, relatively little has been shown about how albatrosses find their food in the open ocean [2], [3]. In general, seabirds find their food by direct visual or olfactory detection or by sighting other predators already feeding on prey. For example, tropical seabirds [4] or Pacific diving ducks [5] have been shown to associate with subsurface predators such as tunas/dolphins or gray whales in order to find and feed on their prey; however, such associations with subsurface predators have not yet been well studied for ocean-going albatrosses. Previous observations of albatrosses from research ships suggest that albatrosses can detect their prey using olfactory cues [6] or by using other albatrosses as visual foraging cues [7], [8]: however, these studies have all had difficulties in following individual birds and thereby in documenting all the foraging strategies employed by individual birds. A recent study on fine-scale movement and prey capture, using GPS and stomach-temperature loggers, suggests that wandering albatrosses, *Diomedea exulans*, may detect prey via olfactory cues in nearly half of their prey-capture events [9]. Nevertheless, fine-scale visual information from the immediate environment around foraging albatrosses should help provide new and additional insight into how albatrosses find their prey.

Recently developed animal-borne image recorders are powerful tools that can obtain visual information from the environment around foraging animals [10], [11]. Although ‘video-tracking’ has been suggested as more effective [11], [12], the relatively large size of devices (but see Rutz *et al*. [13]) and their relatively limited recording duration are problematic for applications that study far ranging animals such as albatrosses. Time-lapse recording of still-images should therefore provide a useful alternative to video, since it has the potential to record the immediate environment around the animals over much longer time periods, yet maintains a relatively small device size. Here, we report on the first use of a still-image camera combined with depth and external temperature data loggers, to study the fine-scale interactions between albatrosses and their environment during their foraging trips over the Southern Ocean.

## Materials and Methods

### Ethics statement

The fieldwork was approved by British Antarctic Survey and the University of Cambridge Animal Ethics Board.

### Methods

The study was conducted at the breeding colony of black-browed albatrosses, *Thalassarche melanophrys*, at Bird Island (54°00'S, 38°03'W), South Georgia, in January 2009. We captured albatrosses at their nest sites and deployed a still-camera system on the back feathers of four birds with Tesa© tape; deployments recorded environmental information over a single foraging trip during the chick-guarding period. We attached the camera to the centre back of the birds, so that the camera view was not permanently obscured by the bird's head while the birds were flying. We recaptured three of the four birds on their nests and retrieved the instruments. The fourth bird could not be recaptured as the bird entered the post-guard chick-rearing stage of breeding during the 3–4 day deployment; during this stage albatrosses visit the nest only briefly (normally for less than 10 min.) after a foraging trip, which makes their recapture very difficult. The fourth camera device was almost certainly lost at sea during subsequent foraging trips while the bird continued to feed its chick.

The camera, manufactured by Little Leonardo Co. Ltd, Tokyo, Japan, had depth and temperature sensors, and was 22 mm in diameter, 132 mm in length, and weighed 82 g in air (<2.7% of the body mass of our study birds). The camera recorded depth and external temperature every second, and captured still-images (1280×1024 pixels) every 30 s with automated exposure time adjustments. The camera had sufficient memory capacity for up to 10,000 images (approximately).

After the recovery of the loggers, image, depth and temperature data were downloaded to a PC. The environment around the study birds was visually inspected in each image, with the appearance of other animals or objects carefully recorded. Depth data were analyzed with Ethographer [14], a behaviour analysis program developed in Igor software (Wave Metrics, USA). Maximum dive depth was calculated for each dive >0.5 m.

## Results

A total of 28,725 images were collected from the three black-browed albatrosses, which covered most (92–99%) of the three foraging trips (duration = 1.9, 3.2 and 4.4 days); foraging trip durations at this stage of breeding for uninstrumented birds range between 0.5 days and 5.5 days [15]. From the archive of images, 12,815 (45% of all images) provided unobscured view of the environment in front of the birds, 9,234 images (32%) were too dark to identify any objects (these were taken mostly during the nighttime) and 6,676 images (23%) were obscured by feathers fluttering in front of the camera lens.

**Figure 1:**
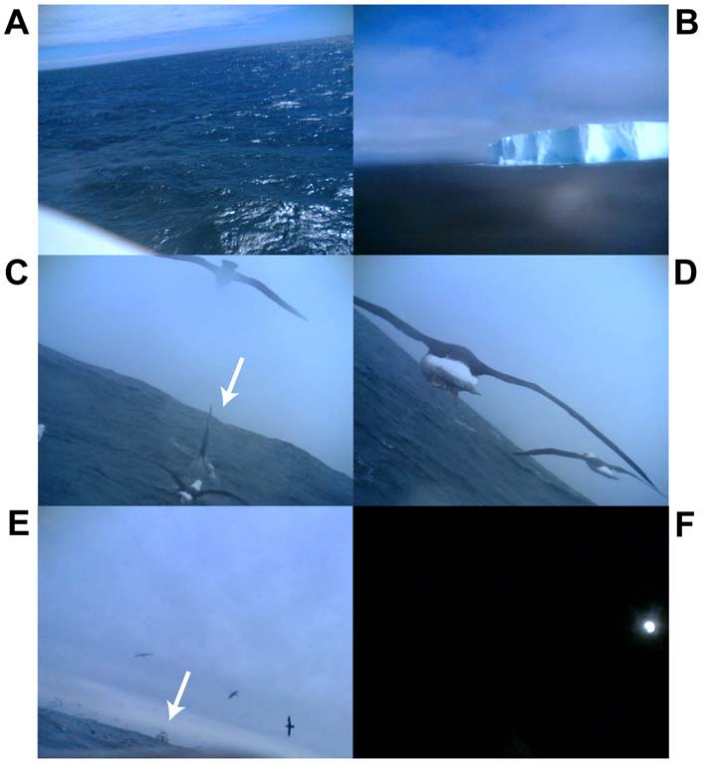
Digital still images obtained from three cameras mounted on black-browed albatrosses. A: a ‘featureless’ sea, B: an iceberg encountered, C: a killer whale breaking the ocean surface, apparent from its dorsal fin (white arrow) and three black-browed albatrosses attracted to the whale, D: two albatrosses flying in association with the camera-mounted bird, E: a fisheries vessel in the distance (white arrow) with an aggregation of birds, F: a bright light source during the night, possibly a vessel or the moon.

**Figure 2:**
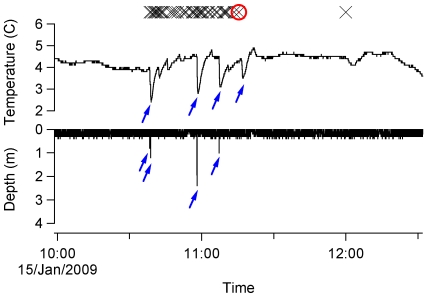
Depth and external temperature profiles from a camera-mounted black-browed albatross. The bird made four dives (>0.5 m: indicated by arrows) over a 2.5-hour period during a foraging trip as indicated by the depth profiles. Rapidly decreasing temperature records were observed in association with dives or possible landing on sea surface (indicated by arrows). The crosses (X) indicate where images of other birds were taken, and the circle indicates where the bird encountered the killer whale (Figure 1C).

Images taken during the daytime mostly showed a ‘featureless’ ocean surface (Fig. 1A). One bird repeatedly encountered relatively large icebergs (Fig. 1B). One surprising finding was that one of the study birds encountered a killer whale, *Orcinus orca*, (Fig. 1C; Fig. 2) during the course of its foraging trip. This image showed that the killer whale broke the surface and that three other albatrosses were also apparently following the whale. This image was, unfortunately, followed by subsequent images that were obscured by feathers. However, the rapidly decreasing external temperature suggests that the bird landed on the sea surface after the encounter with the killer whale (Fig. 2). We do not know how long the bird remained associated with the killer whale, but it potentially continued for over 30 minutes based on the consecutive encounters with other birds and the continuation of the diving activities observed from just before the killer whale was recorded (Fig. 2). Our study birds encountered other birds relatively frequently (in 120 images in total; Fig. 1D). One image showed a vessel, possibly a fisheries vessel, in the distance with an aggregation of seabirds near the vessel (Fig. 1E). Images taken during the nighttime were mostly too dark, but 3 successive images showed a bright light source in front of the albatross, probably from a vessel, or the moon (Fig. 1F).

Our study albatrosses dived infrequently (1.5 dives/d at sea, on average), and only a total of 14 dives (>0.5 m) were recorded (6, 5, and 3 dives per bird). The mean dive depth and duration was 1.46 m and 3.1 s (4.1 m and 11 s for the maximum values). Four (including the 4.1 m dive) out of the 14 dives occurred after the time of sunset. The images obtained before or after these dives showed that it was nearly dark (three dives) or completely dark (one dive). During the daytime, albatrosses tended to dive in association with other birds, as shown by the depth record (Fig. 2) and the associated images; 7 out of 10 daytime dives had associated images of other birds (e.g. Fig. 1D) within 2 minutes either before or after the dive.

## Discussion

This study provides new insights into the interactions between albatrosses and killer whales, an important top predator in the Southern Ocean. The images from our albatross-borne camera show at least four albatrosses (including the camera-mounted bird) actively following a killer whale while it was breaking the sea surface. Only a few previous studies have documented the association between albatrosses and killer whales, and these were mostly in shallow waters (<20 m depth) where some groups of killer whales regularly hunt penguins or pinnipeds along the coast [16]. Previous reviews on the association between seabirds and cetaceans have not reported any records of interactions involving killer whales [17], [18]. However, Hodges and Woehler [19] noted a ship-based observation of one black-browed albatross, among 6 other species of seabirds, flying over a pod of killer whale in the Southern Indian Ocean. Although it is still difficult to quantify how often black-browed albatrosses associate with killer whales in the open ocean, our results, together with ship-based observations such as those of Hodges and Woehler [19], suggest that these associations may occur more frequently than previously anticipated and may be a part of foraging repertoire of albatrosses.

Killer whales occur regularly over the continental shelf around South Georgia [20]. The species feeds on a wide range of prey, such as other whales, pinnipeds and penguins, but they are also known to feed on Patagonian toothfish, *Dissostichus eleginoides*, by stripping them from longline fisheries [20]. Black-browed albatrosses feed mainly on squid, fish and krill (reviewed in Xavier *et al*. [21]), but the deep-water toothfish constitutes an important component of their diet in some breeding localities [22]. Patagonian toothfish or other deep-water fish that occur in their diet [21] could be available to shallow-diving black-browed albatrosses only through an interaction with deep-diving predators (from their food scraps) or with commercial fisheries (from offal or bycatch items). When killer whales feed on fish, fragments of prey are often left near the sea surface [23]. These prey fragments could be an important food resource for albatrosses. Scavenging on such prey fragments may be more energetically advantageous than the pursuit and capture of live prey, as such activities can require frequent take-off, landing, and prey handling which may all be energetically costly [24]. Targeting the less-mobile prey fragments may also reduce the number of plunge dives needed to capture a prey item. Therefore, a close association with foraging killer whales would help albatrosses to find food more efficiently in the apparently ‘featureless’ sea, especially in a year when the availability of aggregative prey species (such as Antarctic krill in South Georgia) is low [25]. Such interactions may be quite common and may account for the presence of other prey species such as lamprey *Geotria australis*, parasitizing to their host, Patagonian toothfish [26], in the diet of the closely related grey-headed albatrosses, *Thalassarche chrysostoma* [21].

The diving behaviour of black-browed albatrosses recorded in this study accords well with previous studies on small-sized albatrosses. The only previous study on black-browed albatrosses reported a maximum dive depth of 4.5 m [27], which is close to our maximum record of 4.1 m. Although the dive duration of black-browed albatrosses has not been reported previously, the mean and maximum dive durations in this study (3.1 s and 11 s) is close to similar-sized grey-headed albatrosses (3.6 s and 14 s) [28] or shy albatrosses *Thalassarche cauta* (4.4 s and 19 s) [29]. The short dives (<5 s) probably reflect plunge dives as previously noted [27], [29], but the dive that was recorded that lasted 11 s suggests that black-browed albatrosses occasionally actively pursue prey underwater, as observed in shy albatrosses [29].

Finally, we suggest that further tracking of marine top predators with animal-borne still image loggers should provide more data on the interaction between marine animals and their environment.
